# Improving the flame retardancy of waterborne polyurethanes based on the synergistic effect of P–N flame retardants and a Schiff base

**DOI:** 10.1039/d0ra01230k

**Published:** 2020-03-30

**Authors:** Hui Wang, Xiaosheng Du, Shuang Wang, Zongliang Du, Haibo Wang, Xu Cheng

**Affiliations:** College of Biomass Science and Engineering, Sichuan University Chengdu 610065 PR China scuchx@163.com +86-28-85401296; The Key Laboratory of Leather Chemistry and Engineering of Ministry of Education, Sichuan University Chengdu 610065 PR China

## Abstract

A novel reactive intumescent fire retardant hexa-[4-[(2-hydroxy-ethylimino)-methyl]-phenoxyl]-cyclotriphosphazene (HEPCP), containing both cyclotriphosphazene and Schiff base structures, is successfully prepared. The chemical structures of HEPCP and flame-retardant waterborne polyurethane (WPU) (FR-WPU) were characterized *via*^31^P, ^1^H NMR and FT-IR. Thermogravimetric (TG) analysis showed that HEPCP exhibited excellent thermal stability and produced rich char residue under high temperature compared with the control sample. The Schiff base and cyclotriphosphazene had a synergistic effect on the WPU. Limiting oxygen index (LOI) values of up to 26.7% were recorded; the dripping behavior was simultaneously improved and achieved a V-1 rating in the UL-94 test by incorporating 0.5 wt% phosphorus. In contrast to the pure WPU, the peak heat release rate (pHRR) of the FR-WPU/HEPCP5 decreased by 43.8%. The char residues increased from 0.63% to 6.96%, and scanning electron microscopy (SEM) showed a relatively continuous and membranous substance, with few holes. The results of TGA-FIR, Py-GC/MS and SEM indicated that HEPCP displayed a fire-retardant mechanism in the condensed-phase. In addition, the thermomechanical behaviors and the mechanical properties indicated that both mechanical properties and *T*_gh_ increased.

## Abstract 2

A novel reactive intumescent fire retardant hexa-[4-[(2-hydroxy-ethylimino)-methyl]-phenoxyl]-cyclotriphosphazene (HEPCP), containing both cyclotriphosphazene and Schiff base structures, is successfully prepared.

## Graphical abstract

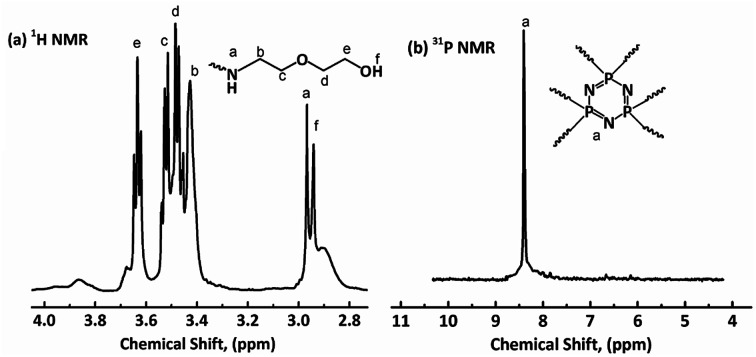

## Introduction

Waterborne polyurethane (WPU) is largely employed in textile laminating, wood coatings, adhesives and leather finishing due to its high chemical resistance, superior mechanical properties, excellent flexibility and remarkable adhesion.^1–4^ However, the high flammability of polyurethane materials limit their application in everyday use.^5–7^ To endow WPU with flame retardant properties, various halogen-free flame retardants have been introduced,^8–11^ among them, phosphorus containing flame retardants have attracted much attention. Typically, the traditional phosphorus-containing flame retardants scavenge the active radicals OH˙ or H˙ in the gas phase, and facilitate dehydration and carbonization in the condensed phase. However, phosphorus-containing flame retardants often decrease the thermal stability of polymers due the low stability of the P–C bond. A common method used to circumvent this issue is to combine phosphorus with nitrogen to prepare intumescent flame retardants (IFRs).^12–15^ Hexachlorocyclotriphosphazene and its derivatives are widely used phosphorus–nitrogen containing flame retardants with high reactivity and excellent fire resistance. They not only improve char forming, but can also enhance the fire resistance and the thermal stability of WPUs due to their synergistic effects with P–N flame retardants.^16,17^ For example, Modesti *et al.*^18^ synthesized two different degrees of –OH substitution cyclotriphosphazene-based flame retardants to prepare flame retardant rigid PU foam. To further enhance the fire resistance of the phosphazene compounds, Yang *et al.*^19,20^ introduced phosphonate and cyclophosphonate to synthesize two reactive cyclotriphosphazene-based flame retardants, and used these compounds to prepare flame retardant rigid PU foam. Although these flame retardants were shown to significantly enhance the fire resistance of a matrix with a high LOI, their limited anti-dripping property is a highly dangerous problem, as it has the potential to lead to secondary fire, fire spread and immediate empyrosis.^21,22^

Schiff base, which is also known as azomethine or imine, is made from the condensation reactions of ketones or aldehydes with primary. Owing to the excellent rigidity of azomethines, Schiff base compounds have been seen as effective in improving the thermal properties of polymers.^23–25^ The azomethine group can also facilitate the crosslinking of melt to enhance the anti-drip properties. Naik *et al.*^26,27^ synthesized *N*,*N*-bis(4-hydroxysalicylidene)ethylene-1,2-diamine and employed it in polyamide-6 fire resistance. The compound was found to promote the formation of an intumescent coating without extra acid sources or synergists, though the total heat release (THR) showed a reduction of only 8%. Wang *et al.*^28–31^ synthesized a series of Schiff base flame retardants and investigated the relationship between Schiff base structure and flame retardancy. They found that Schiff base compounds could effectively improve anti-dripping by promoting the formation of a thermal cross-linkable structure under high temperature and forming a continuous and compact char layer. For this reason, the incorporation of Schiff bases into P–N IFRs can enhance fire resistance and anti-dripping.

In this paper, to improve anti-dripping behavior and the fire resistance of WPU films, a novel reactive inherently flame retardant HEPCP containing cyclotriphosphazene and a Schiff base was designed. The hydroxyl groups of HEPCP were reacted with a diisocyanate prepolymer to obtain polyurethane, which formed WPU dispersion after emulsification. The fire resistance and thermal degradation behaviour of the WPU was studied by UL-94, LOI, cone calorimeter tests, SEM and TGA. The flame-retardant mechanism of HEPCP was studied with a series test. Finally, the thermomechanical behaviours and mechanical properties were studied using DMA analysis and tensile testing.

## Experiments

### Materials

Hexa-phenoxyl-cyclotriphosphazene (HPCP) and 2-(2-aminoethoxy) ethanol were provided by Chengdu Huaxia chemical Reagent Co. Ltd. 1,4-Butanediol (BDO), ethanolamine, acetone, triethylamine (TEA), tetrahydrofuran (THF), methanol, ethanol, and *p*-hydroxybenzaldehyde were provided by Chengdu Kelong Chemical Co., Ltd. Dibutylbis(lauroyloxy)tin (DBTDL), polypropylene glycol-2000, and isophorone isocyanate (IPDI) were provided by Fujian Jinjiang Longzhizu Company. 2,2-Bis(hydroxymethyl)propionic acid (DMPA) were provided by American GEO Specialty Chemicals. Poly(propylene glycol) 2000 (PPG 2000, *M*_n_ = 2000 g mol^−1^) were used after dehydration at 125 °C under 1–2 mm Hg for 3 h. The BDO and DMPA were handled in a vacuum at 100 °C for 12 h.

### Instrumentations

The Fourier transform infrared (FT-IR) spectroscopy was recorded by a Nicolet 560 FT-IR spectrometer (Nicolet, America) with a scanning range from 4000 to 600 cm^−1^. The nuclear magnetic resonance (^1^H and ^31^P NMR) spectroscopy analysis were analyzed by a Bruker AV400 NMR spectrometer (Bruker, US), and the used solvent was Dimethyl Sulfoxide-d_6_ (DMSO-d_6_). Thermogravimetric analysis (TGA) was measured on a TG 209 F3 Tars (Netzsch, Germany) by heating from 50 °C to 600 °C under nitrogen atmosphere. The heating rate was 10 °C min^−1^ the LOI test was conducted by a HC-2C oxygen index meter (Jiangning Analysis Instrument Company, China) according to the ASTM-D 2863-97 with a dimension of sample was 130 × 6.5 × 2.7 mm^3^. The vertical burning test (UL-94 test) was measured according to ASTM D3801-2010 using CZF-2 vertical burning instrument (Jiangning Analysis Instrument Company, China). The sizes of the specimens were 130 × 6.5 × 2.7 mm^3^. The cone calorimeter testing (CCT) was measured according to the ISO 5660-1 standard on a cone calorimeter (Fire Testing Technology, UK). The sizes of the samples were 100 × 100 × 3 mm^3^. The spectroscopy of TG-IR was analyzed by TGA Q5000 thermal analyzer coupled with a Nicolet 10 FT-IR spectrometer. 5–10 mg samples were tested at the temperature from 50 to 600 °C under N_2_ with the heating rate of 20 °C min^−1^ under N_2_ atmosphere. The pyrolysis behavior of specimens was conducted by using a CDS Pyroprobe 5000 instrument and chromatograph mass spectrometer GC-MS. The cracker temperature was 500 °C. And the generated gases were separated and detected by MS.

The morphologies and the element content of char residues from CCT were performed by a FEI Quanta 250 scanning electron microscope and energy dispersive X-ray spectrometry (EDX) analyzer. The Laser Raman test was performed on a laser Raman spectrometer (SPEX-1403, America) at room temperature to characterize the char residues after the CCT tests with a scanning range from 200 cm^−1^ to 2000 cm^−1^ and the excitation wavelength was 532 nm. The mechanical properties of FR-WPU films were conducted according to ASTM D 638 standard using a UTM 6203 electronic universal testing machine (Shenzhen SANS Test Machine Co. Ltd., China). The dynamic mechanical thermal analysis (DMA) was conducted by using a TA Dynamic Mechanical Thermal Analyzer Q800 (TA Instruments). The samples were measured from −80 °C to 120 °C with a heating rate of 3 °C min^−1^.

### Synthesis of hexa-[2-(2-hydroxy-ethoxy)-ethylamino]-cyclotriphosphazene (HDPCP)

**Scheme 1:**
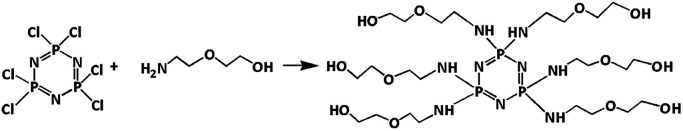
The synthetic route of HDPCP.

The synthesis of HDPCP was according to the procedure literature,^32^ as illustrated in Scheme 1. A four-necked glass reactor equipped with thermometer, mechanical stirrer, condenser tube, and nitrogen inlet, 1.05 g (6.0 mmol) HCCP and 500 mL THF were added at room temperature. Subsequently, a mixed solution of 40 mL triethylamine and 7.2 g (68.5 mmol) 2-(2-aminoethoxy) ethanol dissolved in absolute THF (300 mL) were dropwise added into the reactor. And the reaction was stirred at room temperature for 12 hours under nitrogen, then the reaction was kept at 40 °C for another 12 h. Then, the reaction temperature was decreased to room temperature, and the THF was removed by evaporation. The resulting crude product was washed three times by a solution of methanol–THF (1 : 5). Finally, a pale-yellow sticky liquid product was obtained by drying in a vacuum oven at 100 °C for 24 h, and its yield was 76.3%.

### Synthesis of hexa-[4-[(2-hydroxy-ethylimino)-methyl]-phenoxyl]-cyclotriphosphazene (HEPCP)

**Scheme 2:**
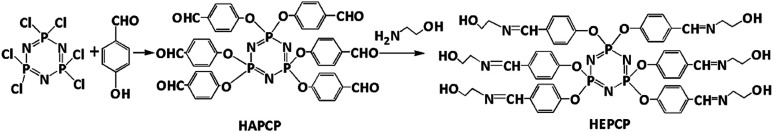
The synthetic route of HEPCP.

The synthesis of the Schiff base coupling of cyclotriphosphazene (HEPCP) was prepared *via* a two-step prepare methods,^33,34^ as illustrated in Scheme 2. First, the intermediate (HAPCP) was synthesized in a four-necked glass reactor equipped with thermometer, mechanical stirrer, condenser tube, and nitrogen inlet. 20 g (0.16 mol) *p*-hydroxybenzaldehyde, 8.35 g (0.024 mol) HPCP, 34.55 g (0.25 mol) K_2_CO_3_, and 100 mL THF were added into the necked glass reactor at room temperature. Then heat the reaction temperature to 65 °C, and continue stirring for 24 h under N_2_ atmosphere. The crude product was obtained after the evaporation of the solvent THF. Then the resulting product was recrystallized and purified by ethyl acetate, and the yield of product was 93.1%.

Secondly, a four-necked glass reactor equipped with thermometer, mechanical stirrer, condenser tube, and nitrogen inlet, the obtained intermediate (HAPCP) 17.2 g (0.02 mol), ethanolamine 8.6 g (0.14 mol) and ethanol (300 mL) were added into the reactor. The reaction was stirred at 50 °C for 4 hours. After reaction, the THF was removed by evaporation. The crude product was washed with ethanol three times and dried in a vacuum oven for 24 h at 80 °C. Then the slight yellow solid product was obtained and the yield was 91.5%.

### Preparation of FR-WPU

**Scheme 3:**
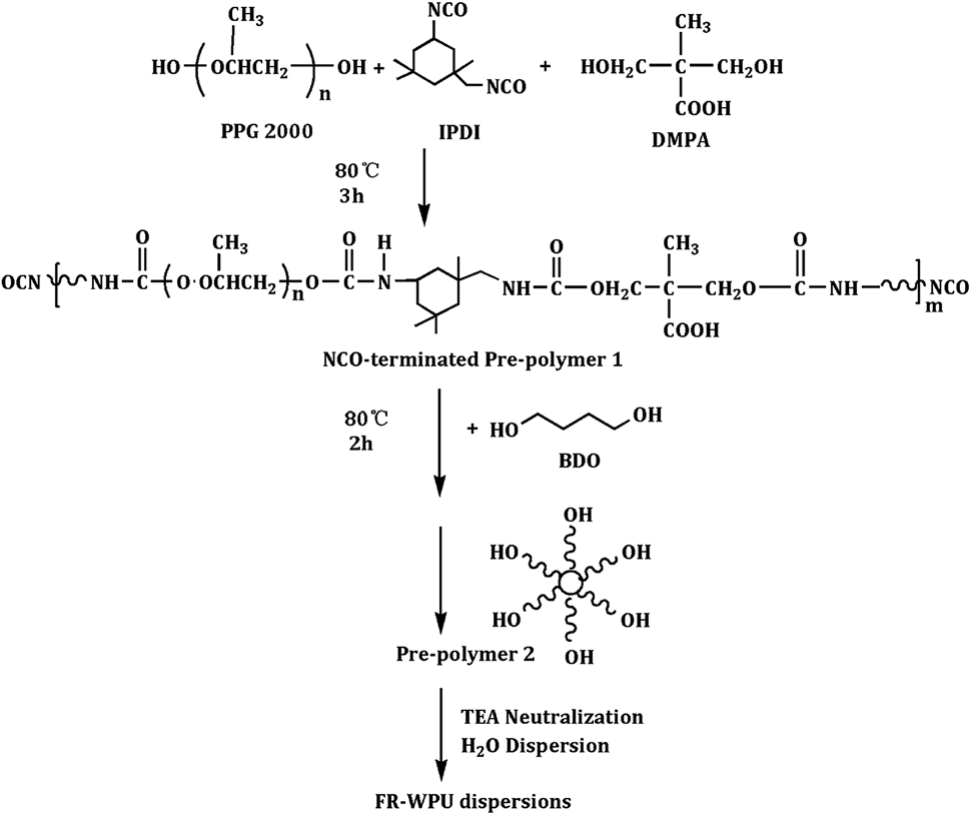
The synthetic route of FR-WPU dispersions.

According to the previously reported method,^35^ a series of the FR-WPUs were prepared as the following steps: firstly, 22.23 g IPDI, 3.19 g DMPA, 34.2 g PPG 2000, and a few drops catalyzer were put into the three-neck flask equipped with condenser tube and mechanical stirrer. The mixture was stirred at 85 °C for 2 h. Then, different quantity of BDO was added into the reactor for another 2 h. Afterwards, different quantity of polyols was put into the flask until the calculated value of –NCO was achieved. The value of NCO was measured by the standard dibutylamine back-titration method. After reaction, the temperature dropped to about 40–45 °C. Subsequently, the mixture was neutralized by TEA dissolved in acetone and stirred at 45 °C for 30 min, followed by dispersion under vigorous stirring with deionized water. Finally, a FR-WPU emulsions were obtained after evaporation of acetone, in which has a solid weight of 32 wt%. The recipes to prepare FR-WPU with different molecular weight and their chemical compositions were presented in Table 1. The reaction scheme of modified WPU emulsions was showed in Scheme 3. The modified WPUs in this paper were marked as pure WPU, FR-WPU/HDPCP5 and FR-WPU/HEPCP5, where 5 indicated the phosphorus contents in WPU were 5‰.

**Table 1:** The chemical composition of pure-WPU and FR-WPU dispersions

Sample	PPG-2000	HDPCP	HEPCP	DMPA	BDO	IPDI	TEA
Pure-WPU	30.00	—	—	2.80	2.55	19.50	2.10
FR-WPU/HDPCP5	30.00	2.44	—	2.80	1.13	19.50	2.10
FR-WPU/HEPCP5	30.00	—	3.60	2.80	1.13	19.50	2.10

### Preparation of FR-WPU films

The calculated dose of FR-WPU emulsions was added into the Teflon bath for 5 d at 25 °C. Then, the FR-WPU films were dried at 60 °C for 2 d to eliminate the remaining water.

## Results and discussion

### Characterization of HDPCP, HEPCP and FR-WPU

**Fig. 1:**
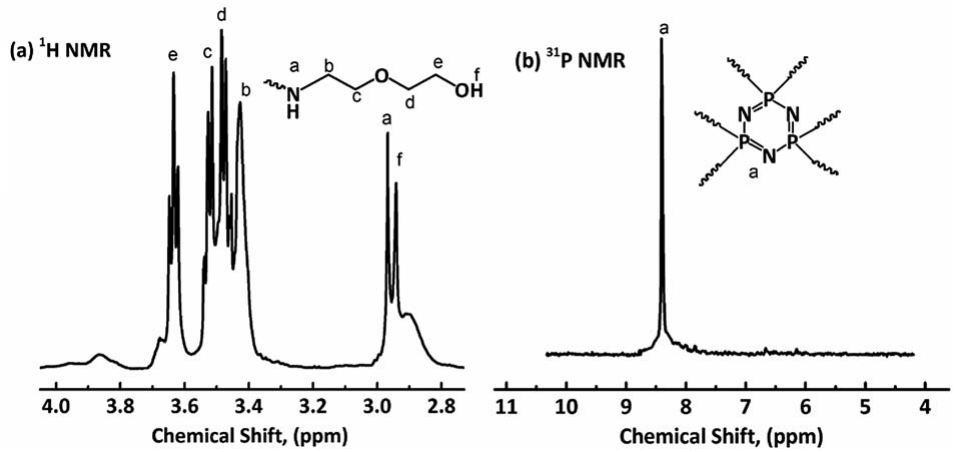
NMR spectra of HDPCP: (a) ^1^H NMR; (b) ^31^P NMR.

**Fig. 2:**
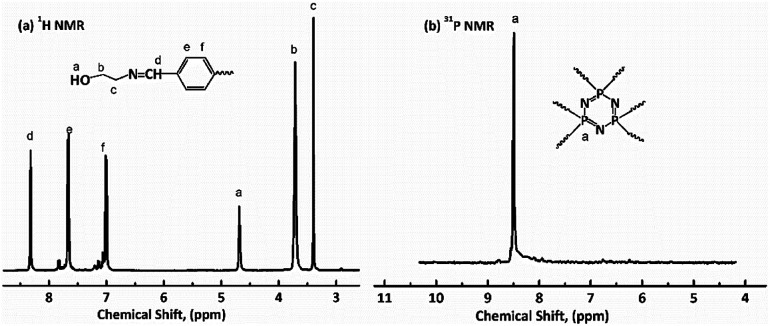
NMR spectra of HEPCP: (a) ^1^H NMR; (b) ^31^P NMR.

^1^H NMR, ^31^P NMR and FT-IR were used to characterize the chemical structures of HDPCP and HEPCP, and the results are shown in Fig. 1 and 2. The ^1^H NMR and ^31^P NMR spectra of HDPCP are shown in Fig. 1. The signal at 2.90 ppm contributed to the chemical shift of protons in the hydroxyl group and the signal at 2.96 ppm contributed to the chemical shift of –NH–. Signals around 3.50 ppm contributed to the chemical shift of methylene attached to –O–, signals around 3.42 ppm contributed to the protons of –CH_2_– attached to –NH, and the signal at 3.63 ppm contributed to the protons of –CH_2_– attached to the –OH. These results were similar to the results found in previous reports.^32^Fig. 1(b) shows a signal at 8.41 ppm in the ^31^P NMR spectrum of HDPCP.

The ^1^H NMR spectrum of HEPCP is shown in Fig. 2(a). The peak signal at 3.40 ppm contributed to the –CH_2_– linked with –N=CH–, the signal at 3.71 ppm contributed to the protons of –CH_2_– attached to –OH, the signal at 4.60 ppm was attributable to the chemical shift of protons of hydroxyl group, the chemical shifts at 7.01 and 7.65 ppm contributed to the aromatic protons, and the signal at 8.36 ppm contributed to the protons of –N=CH–. Similar to HDPCP, the ^31^P NMR spectrum of HEPCP showed only a peak at 8.49 ppm, as shown in Fig. 2(b). These results indicate that the target products of a Schiff base and cyclotriphosphazene structure containing a polyol were successfully synthesized.

**Fig. 3:**
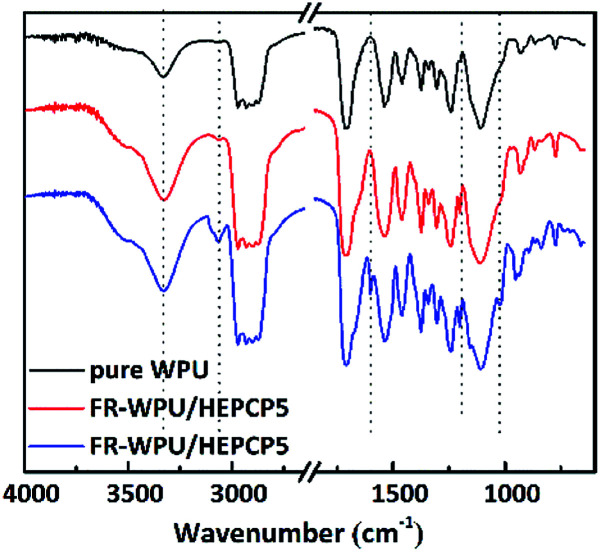
FT-IR spectra of pure WPU, FR-WPU/HDPCP5 and FR-WPU/HEPCP5.

The synthesized FR-WPU/HDPCP5 and R-WPU/HEPCP5 films were characterized by FT-IR. As shown in Fig. 3, FT-IR showed a broad absorption signal at 3332 cm^−1^ of WPU and was assigned to the –NH– stretching vibration of the urethane bond. The signal at 1533 cm^−1^ referred to –NH– deformation and the signal at 1711 cm^−1^ contributed to the –C=O bonds. The peaks from 2978 cm^−1^ to 2869 cm^−1^ contributed to the symmetrical and asymmetrical stretching absorption bands for the –CH_3_ and –CH_2_– groups. Both the FR-WPU/HDPCP5 and FR-WPU/HEPCP5 films showed characteristic infrared absorption signals at 1203 cm^−1^, which contributed to –P=N– stretching absorption bands. For FR-WPU/HEPCP5, the newly generated signals at 3070 cm^−1^ and 1598 cm^−1^ contributed to the Ar–H and the –N=CH– stretching vibrations, respectively. The signal at 1022 cm^−1^ contributed to P–O–Ar. These results confirmed the successful synthesis of FR-WPU/HDPCP5 and FR-WPU/HEPCP5.

### Thermal properties

**Fig. 4:**
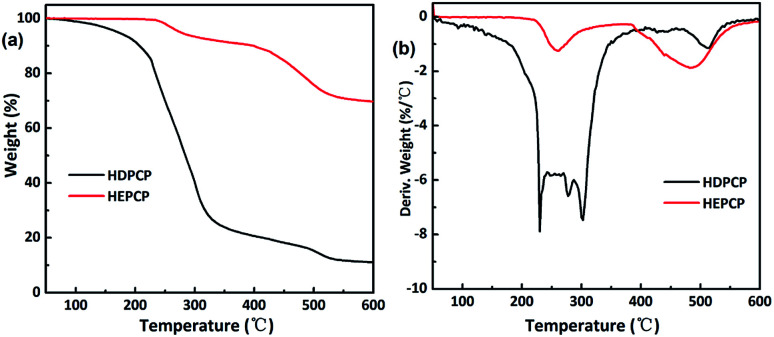
TGA and DTG curves of HDPCP, HEPCP: (a) TGA curves; (b) DTGA curves.

The TGA and differential thermogravimetric (DTG) curves of the HDPCP and HEPCP under N atmosphere are shown in Fig. 4(a) and (b), respectively. Data for the temperature at the maximum decomposition rate (*T*_max_), the initial decomposition temperature of 5% weight loss (*T*_5%_) and the residue yield at 600 °C gained from the TGA curves are listed in Table 2. For HDPCP, the *T*_5%_ occurred at 171 °C. A major mass loss *via* a great decrease of approximately 70 wt% from 170 °C to 340 °C was observed, which may be attributable to the relatively low stability of the P–N bond. As shown in Fig. 4, HEPCP exhibited less weight loss than HDPCP from 170 °C to 340 °C, and its residue char reached 69.66 wt% at 600 °C, indicating that the synthesized HEPCP had char forming control under high temperatures. This is likely due to the large amount of cyclotriphosphazene present, as well as its aromatic structure. Moreover, there was crosslinking of the azomethine linkage at higher temperatures, which also led to less weight loss.

**Table 2:** TGA and DTG results for HDPCP, HEPCP and FR-WPU films

Sample	*T*_d5%_ (°C)	*T*_max1_ (°C)	*T*_max2_ (°C)	*T*_max3_ (°C)	*T*_HRI_ a (°C)	Residues (wt%)
HDPCP	171.16	229.40	299.93	517.43	108.10	11.03
HEPCP	172.43	254.52	485.02	—	204.82	69.66
Pure-WPU	262.14	329.25	373.82	—	143.95	1.55
WPU/HDPCP5	255.61	334.66	374.93	—	145.06	3.29
WPU/HEPCP5	242.73	269.63	327.13	386.08	143.86	7.24

a *T*_HRI_ = 0.49 × [*T*_5%_ + 0.6 × (*T*_30%_ − *T*_5%_)]. Where *T*_5%_ and *T*_30%_ is corresponding decomposition temperature of 30 wt% weight loss, respectively.

**Fig. 5:**
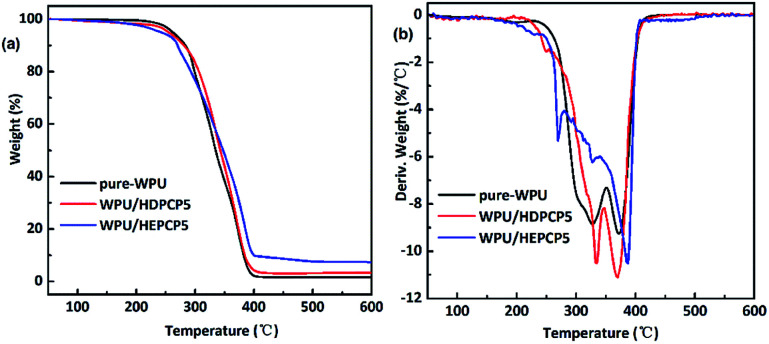
TGA and DTG curves of the pure WPU and FR-WPU films: (a) TGA curves; (b) DTGA curves.

The degradation and char forming behavior of the FR-WPU/HDPCP5 and FR-WPU/HEPCP5 films under a nitrogen atmosphere was studied using TGA tests. The results are given in Fig. 5 and Table 2. The temperature at a weight loss of 5% (*T*_d5%_), the temperature at the maximum decomposition rate (*T*_max_), the residual at 600 °C and the calculated heat-resistance index (*T*_HRI_)^36^ are summarised in Table 2. As can be seen from Table 2, there are no significant changes of *T*_5%_, the *T*_HRI_ and residual char remaining at 600 °C increased significantly. As described in previous reports, the degradation behavior of polyurethane mainly occurred in two phrases under nitrogen. In case of pure WPU, decomposition occurred between 251.34–350.41 °C and 350.41–426.82 °C, and *T*_max_ at 347.40 °C and 387.02 °C. During the degradation process, the urethane bonds were broken in the hard segment and there was further decomposition of the soft segment.^37^ Additionally, there was only 1.55% residual char remaining at 600 °C. After the introduction of flame retardants, there was a similar degradation trend in the modified FR-WPU. The introduction of HDPCP into FR-WPU was found to decrease the initial decomposition temperature, and increase *T*_max1_, which was most likely due to the lower stability of the P–N bond at this decomposition temperature range. The fast decomposition of the P–N linkage promoted the degradation of the matrix to form char during the heating process by forming poly(phosphoric) acids. In contrast, the introduction of HEPCP into FR-WPU decreased *T*_5%,_*T*_max1_ and *T*_HRI_, contributing to HEPCP's lower thermal stability compared with WPU. Although the *T*_5%_ and *T*_HRI_ of FR-WPU/HEPCP5 is lower than that of FR-WPU/HDPCP5, it is worth noting that the decrease extent of the *T*_HRI_ reduces significantly. It showed that the thermal stabilities of the FR-WPU/HEPCP5 was improved with the increasing temperature.^38^ The second step of the thermal degradation was caused by the urethane bond in WPU, and the third step was caused by the thermal decomposition of the soft segments and cyclotriphosphazene moieties.^36^ Compared to the other two types of WPU, the FR-WPU/HEPCP5 had a higher *T*_max_ ranging 335–390 °C. The degradation temperature *T*_max2_ of FR-WPU/HEPCP5 was similar to the *T*_max1_ of pure WPU, though the *T*_max3_ of FR-WPU/HEPCP5 was significantly increased. This could be due to the thermally induced crosslinking action caused by the azomethine double bond in HEPCP, which could delay the decomposition of WPU.^39,40^ Furthermore, the amount of char residue at 600 °C in FR-WPU/HDPCP5 and FR-WPU/HEPCP5 improved to 3.29% and 7.24%, respectively. WPU/HEPCP5 showed a higher char yield percentage (7.24%) than the WPU/HEPCP5 system (3.29%). This is likely due to the presence of the Schiff base structure and higher aromatic ring content in the WPU matrix.^41^

**Fig. 6:**
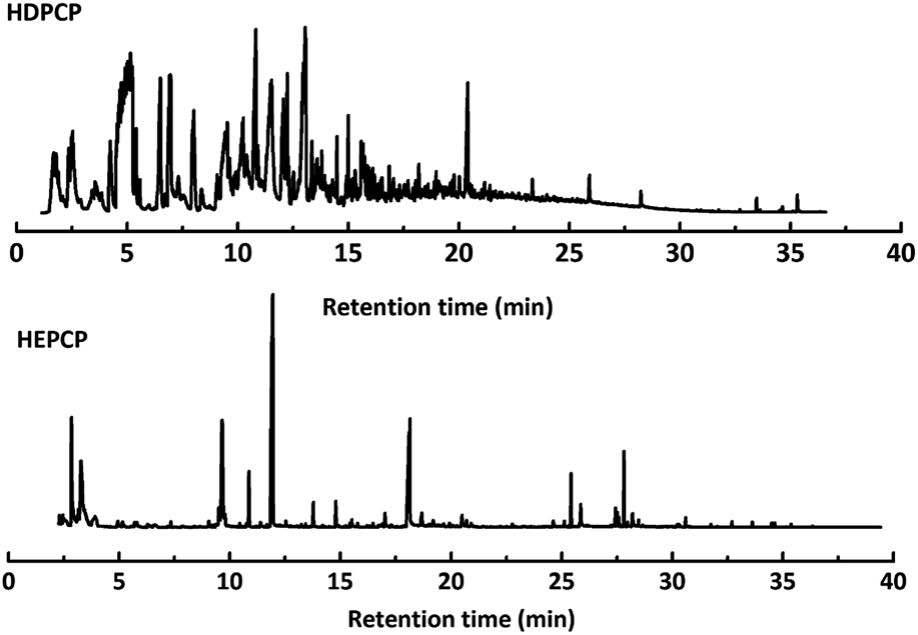
Pyrochromatogram of HDPCP and HEPCP at 500 °C.

Py-GC/MS tests on HDPCP and HEPCP were performed to investigate the products of the thermal decomposition in the gaseous phase (Fig. 6). The pyrolysis products were analyzed at 500 °C, and the results are shown in Table 3. The pyrolysis products for HDPCP and HEPCP were different, but they can all be generally divided into two categories. The first category consists of amine compounds, including morpholine, pyridine and their homologues. The second category consists of aromatic structure compounds and small molecule hydrocarbons. For HDPCP, these fragments arose from the rearrangement reaction and free radical reaction with OH or H free radicals. For HEPCP, the new benzene fragments were produced by the simple crack and rearrangement of HEPCP. As effective nucleating agents, benzene fragments can be aggregated to form smoke particles.^42^ The compounds containing nitrogen atoms may be derived from the rearrangement and cyclizing reaction between azomethine and the linked benzene fragment. Significantly, phosphorous-containing compounds were not monitored in the pyrolysis for both HDPCP and HEPCP at 500 °C, indicating that cyclotriphosphazene remained in the residue. Therefore, HDPCP and HEPCP were shown to have similar effects in the solid phase.

**Table 3:** Pyrolysis products identified in the pyrograms of HDPCP and HEPCP

Retention (min)	*m*/*z*	Assigned structure	Retention (min)	*m*/*z*	Assigned structure
2.544	79	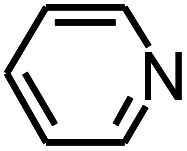	2.844	87	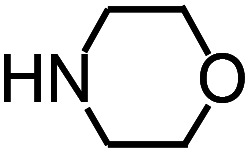
4.23	101	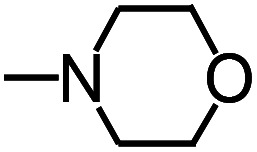	3.259	79	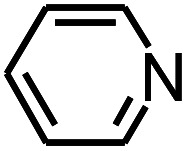
4.67	87	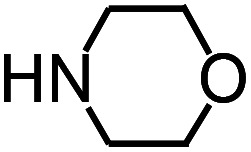	3.895	92	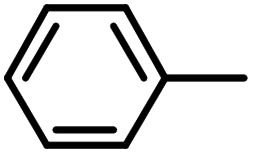
4.725	131	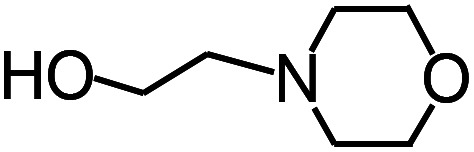	9.512	93	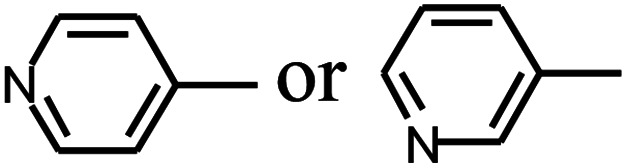
6.506	93	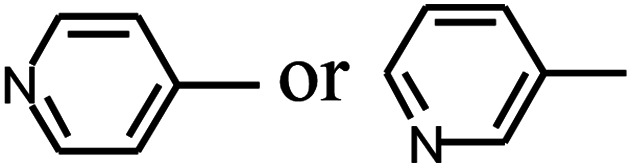	9.652	94	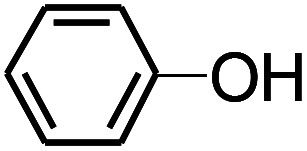
6.916	115	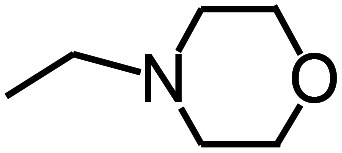	10.863	136	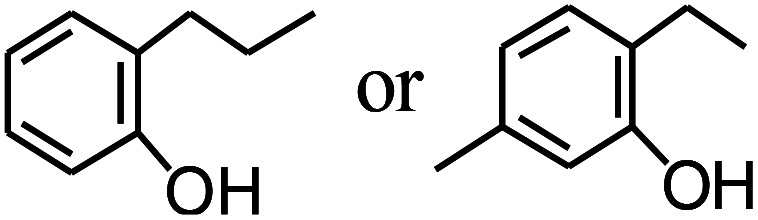
7.321	170	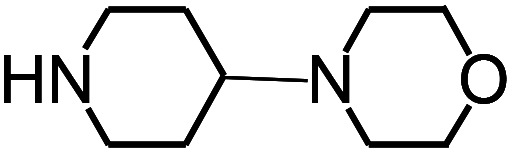	11.943	108	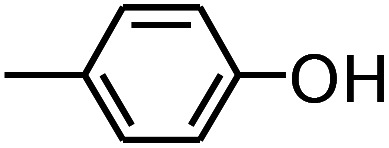
9.437	122	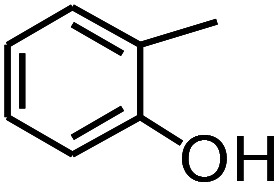	14.794	120	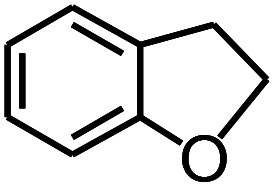
10.827	121	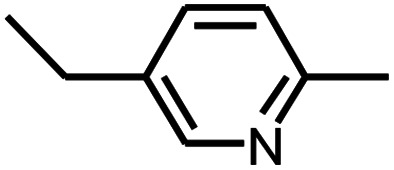	18.14	122	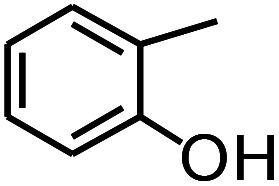
11.533	173	C_8_H_15_O_3_N	25.433	270	C_17_H_34_O_2_
13.053	150	C_9_H_14_N_2_	25.869	256	C_16_H_32_O_2_
20.401	164	C_10_H_16_N_2_	27.819	298	C_19_H_38_O_2_

**Fig. 7:**
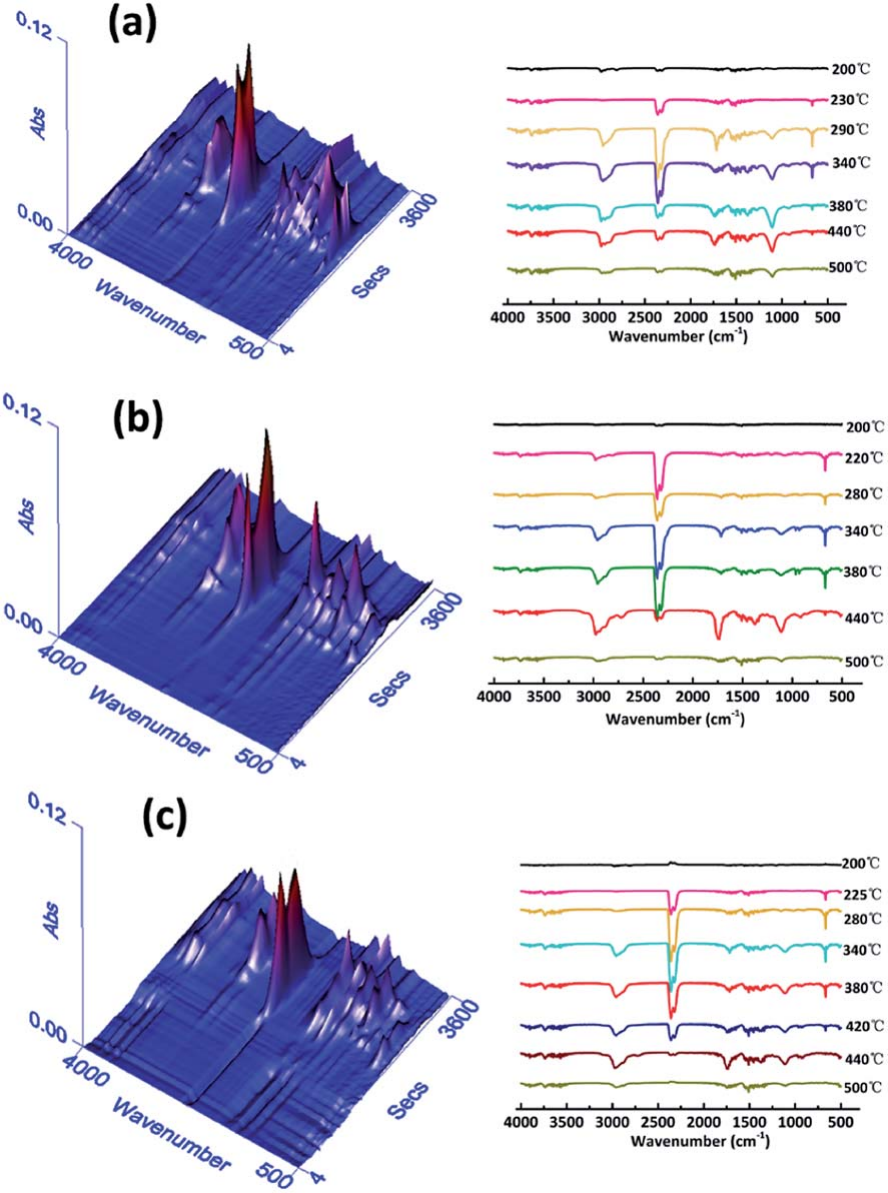
The 3D TG-IR spectra and the TG-IR spectra at different temperature of pure WPU (a), FR-WPU/HDPCP5 (b) and FR-WPU/HEPCP5 (c).

TG-IR is usually used to study the volatile gasses produced during thermal degradation. Fig. 7 presents the 3D TG-IR spectra of pyrolysis products and the FT-IR spectra of pyrolysis gasses under different degradation temperatures for pure WPU, FR-WPU/HDPCP5 and FR-WPU/HEPCP5. There were no obvious differences in the varieties of volatile products for pure WPU, FR-WPU/HDPCP5 and FR-WPU/HEPCP5. In the curves of the three samples, there were characteristic signals of gases containing –OH, such as water (3620–3780 cm^−1^) and hydrocarbons (2828–2998 cm^−1^), gases containing CO_2_, –NCO groups and HCN (2358–2324, 668 cm^−1^), carbon monoxide (1718 cm^−1^) and ethers (1100 cm^−1^), NH_3_ (964 cm^−1^),^13,31,43^ and the characteristic bands were mostly similar. The signal intensity changed with increasing temperature. In the case of pure WPU, gases containing CO_2_, –NCO groups and HCN were released at 220–490 °C. Peak intensities increased to the maximum (about 475 °C) and then decreased gradually with the temperature. However, for FR-WPU/HDPCP5 and FR-WPU/HEPCP5, the pyrolysis products were released earlier than pure WPU. This may be due to the acceleration of the decomposition of FR-WPU by HDPCP or HEPCP at relatively low temperatures. In addition, the peak intensities for FR-WPU/HDPCP5 and FR-WPU/HEPCP5 were weaker than that of pure WPU when the products were obtained at higher temperatures. Notably, the peak intensities of the pyrolysis products of FR-WPU/HEPCP5 films were weaker than those of FR-WPU/HDPCP5, suggesting that HEPCP has a better barrier effect on decomposed gas release than HDPCP*via* the accumulation of thermal-stable char layers. However, it is worth pointing out that phosphorus-containing gas products were not detected in the FT-IR spectra of WPU/HDPCP5 and FR-WPU/HEPCP5 in the whole degradation process. During the degradation process, the interaction between WPU and flame retardants occurred in the solid phase, and the phosphorus-containing compounds remained in the solid phase. Therefore, the fire retardants catalyzed the formation of the char layer, which inhibited the transfer of the pyrolysis gas products and O_2_. The results demonstrate that HDPCP and HEPCP mainly displayed a condensed phase mechanism, which is consistent with the Py-GC/MS results discussed earlier.

### Fire resistance

The LOI test and UL-94 vertical burning test are two common methods used to determine the flammability of polyurethane films. The flammability of pure WPU, FR-WPU/HDPCP5 and FR-WPU/HEPCP5 was investigated, and the data are showed in Table 4. The LOI values of pure WPU, FR-WPU/HDPCP5 and FR-WPU/HEPCP5 were 18.4%, 26.2% and 26.7%, respectively. In comparison with pure WPU, the LOI values of the FR-WPU/HDPCP5 and FR-WPU/HEPCP5 films were both improved. The LOI value of the FR-WPU/HEPCP5 film was similar to the FR-WPU/HEPCP5 film, indicating that there were no significant differences in LOI values when Schiff base structures were introduced into the WPU structures.

**Table 4:** LOI values and UL-94 of different FR-WPU films

Sample	LOI (%)	UL-94 vertical burning test		
		UL-94 rating	Dripping	Ignition the cotton
Pure-WPU	18.4	No rating	Yes	Yes
WPU/HDPCP5	26.2	V-2	Yes	Yes
WPU/HEPCP5	26.7	V-1	Yes	No

As depicted in Table 4, the results of the UL-94 test revealed a significant difference. The FR-WPU containing HDPCP had a V-2 UL-94 flammability rating, whereas the FR-WPU containing HEPCP had a V-1 UL-94 flammability rating. Although the incorporation of HEPCP and HEPCP did not eliminate the melt dripping of WPU, FR-WPU/HEPCP5 dripping without a flame cannot ignite cotton, and the reduction in the degree of dripping during the test compared to the FR-WPU/HDPCP5. This is likely due to the Schiff base structures and the higher weight percentage of the benzene rings in the polyurethane molecules. During combustion, HEPCP promoted the formation of an intumescent char layer and enhanced the melting viscosity through a crosslinking action. According to the TGA results, HEPCP exhibited a higher thermal stability under high temperature and formed a rich char. The residual char is able to insulate the heat, prevent flame propagation and reduce smoke generation.

**Fig. 8:**
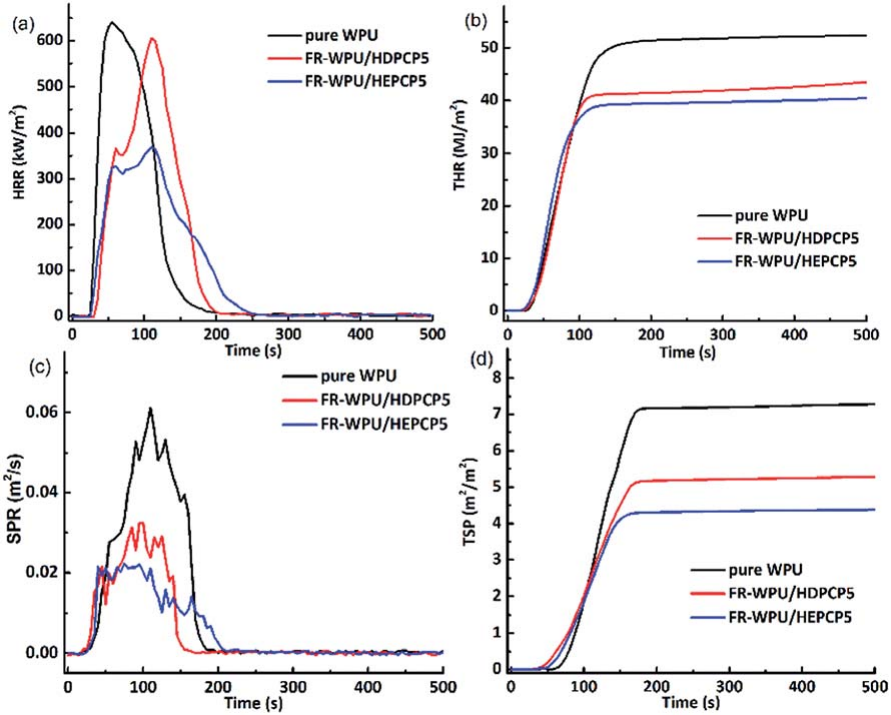
The HRR, THR, SPR and TSP curves of pure WPU, FR-WPU films after cone calorimeter test: pure WPU; WPU/HDPCP5; WPU/HEPCP5.

The flame retardancy of pure WPU, FR-WPU/HDPCP5 and FR-WPU/HEPCP5 films were evaluated using the cone calorimeter test (CCT) to simulate the combustion process in a real fire scenario. The typical parameters, including the heat release rate (HRR), the time to ignition (TTI), total smoke production (TSP), total heat release (THR), yield of char residue and smoke production rate (SPR), the curves of HRR, SPR, TSP, THR and the corresponding data are shown in Table 5 and Fig. 8. The HRR curve showed a single sharp peak. Pure-WPU was found to be highly flammable. It burned rapidly after being lit with a peak heat release rate (pHRR) of 640.48 kW m^−2^ that appeared at 55 s. The THR of pure WPU was 52.32 MJ m^−2^. When HDPCP or HEPCP was incorporated into WPU, the TTI was delayed compared with pure WPU, as shown in Table 5. This contributed to the formation of a protective char coating during the burning process. There was a similar trend in HRR and THR with pure WPU, but the values of pHRR and THR decreased when HDPCP or HEPCP was introduced to the WPU. They also prolonged the burn time of pHRR. The values of pHRR and THR decreased by 6.7% and 43.8%, respectively. Considered together, the results of TG, HRR and THR indicate that HDPCP or HEPCP can improve the fire resistance properties of FR-WPU.

**Table 5:** Cone calorimetric results and LOI values of FR-WPU

Sample	TTI (s)	*p*-HRR (kW m^−2^)	THR (MJ m^−2^)	SPR (m^2^ s^−1^)	TSP (m^2^ m^−2^)	Residues (wt%)
Pure-WPU	34	640.48	52.32	0.061	7.27	0.63%
FR-WPU/HDPCP5	40	599.75	42.98	0.032	5.27	3.23%
FR-WPU/HEPCP5	41	369.60	40.16	0.022	4.27	6.96%

The TSP and SPR are important factors to understand the smoke emission behaviors during the combustion process of polyurethane materials. Fig. 8(c) and (d) shows the curves of SPR and TSP. By comparison with pure WPU, the introduction of HDPCP or HEPCP significantly reduced the peak SPR and TSP values for FR-WPU/HDPCP5 and FR-WPU/HEPCP5. The TSP value of pure WPU was 7.27 m^2^ m^−2^, whereas those of FR-WPU/HDPCP5 and FR-WPU/HEPCP5 were 5.27 and 4.27 m^2^ m^−2^, which are reduced by 27.5% and 41.3%, respectively. This suggests that both HDPCP and HEPCP had a smoke suppression effect. For FR-WPU/HEPCP5, the Schiff base structure changed the melt viscosity of the WPU and the structure of the char residue, which led to effective smoke suppression.^45^

The aforementioned results suggest that HDPCP and HEPCP decomposed earlier than the polyurethane, and incorporating them decreased the temperature of the onset of decomposition, though they improved the thermal stability at high temperatures. This indicates that incorporating HDPCP or HEPCP has a condensed phase flame retardant effect for mainly on the stronger charring ability and the generation of a protective char layer. The char layer is beneficial as it helps prevent volatile products from transferring to the burning zone and protects the PU matrix from oxygen and heat. Therefore, the results of this study indicate that incorporating DHTBN has beneficial effects on fire resistance in FR-WPU.

**Fig. 9:**
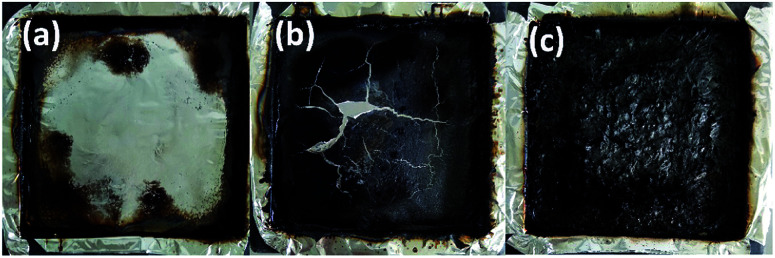
Digital photographs of residues of pure WPU, FR-WPU films after cone calorimeter test: (a) pure WPU; (b) WPU/HDPCP5; (c) WPU/HEPCP5.

**Fig. 10:**
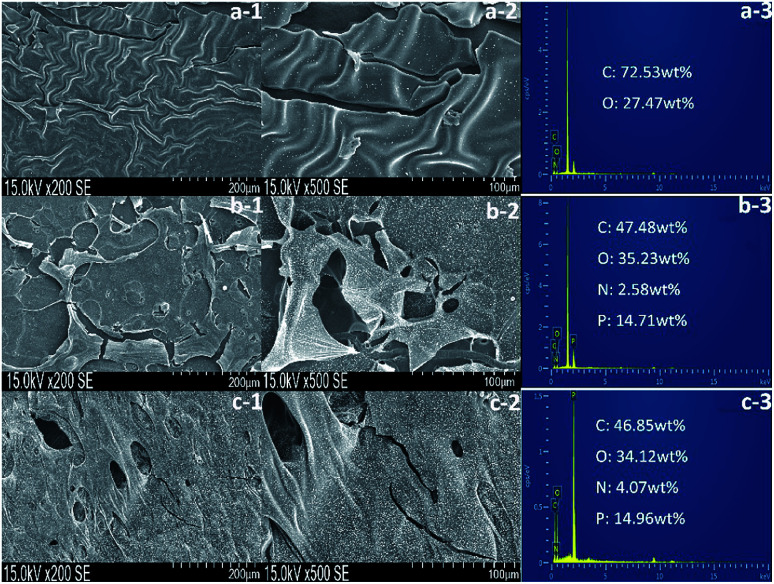
SEM micrographs and of EDS images of pure WPU, FR-WPU/HDPCP5 and FR-WPU/HEPCP5.

The residual char of polyurethane films during the burning process is a critical parameter in flame retardant research. As shown in Table 5, the char residues of pure WPU, FR-WPU/HDPCP5 and FR-WPU/HEPCP5 were 0.63%, 3.23% and 6.96%, which is in keeping with results of TGA. Fig. 9 provides digital photographs of the residues of pure WPU, FR-WPU/HDPCP5 and FR-WPU/HEPCP5 from the CCT. As can clearly be seen, the pure-WPU is almost burnt out in Fig. 9(a), and little residual char remained after burning. Unlike pure WPU, the char residues of flame-retardant treated WPU formed intumescent char. For FR-WPU/HDPCP5, a continuous protective layer was formed. When HEPCP was incorporated, a more compact and continuous residual char for FR-WPU/HDPCP5 was formed, which indicated the existence of a condensed phase flame-retardant mechanism.^45^ Relative to the control sample, the lower pHRR values of FR-WPU/HDPCP5 and FR-WPU/HEPCP5 were attributable to the coverage of the continuous and firm char layers. Fig. 10 shows the morphologies and the element content of residual char of the outer microstructures of pure-WPU, FR-WPU/HDPCP5 and FR-WPU/HEPCP5 from CCT. The char residue of pure WPU was uneven and fragmented with cracks in the local area and could not effectively prevent fire and heat transfer, resulting in poor fire resistance. While the other samples showed significant differences in the surface of the char layers, FR-WPU/HDPCP5 formed an intumescent char with some holes and crevasses on the surface (Fig. 10(b)), indicating that heat, oxygen and flammable gases could transfer through the char layer. In the case of FR-WPU/HEPCP5, the outer residue was a relatively continuous and membranous substance with few holes (Fig. 10(c)). This structure can hinder oxygen, heat and flammable gas migration between the burning zone and the material. The results suggest that the introduction of the cyclotriphosphazene group and Schiff base structures dramatically changed the morphology of the char residues, resulting in the formation of a protective shield on the surface that acted as a physical barrier to hinder heat and oxygen diffusion. Thus, the flame-retardant properties of the WPUs were significantly improving. From Fig. 10, only oxygen and carbon elements were observed in the char residue of the pure WPU. However, a 14.71 and 14.96 wt% phosphorus content were identified in the char residues of FR-WPU/HDPCP5 and FR-WPU/HEPCP5, respectively. This indicates that incorporating HDPCP or HEPCP resulted in a char residue rich in phosphorus elements that can catalyze dehydration and carbonization at higher temperatures. This result is in keeping with the Py-GC/MS analysis. Additionally, we can see the content of nitrogen does not decline during the decomposition of WPU, in contrast, after incorporating HEPCP the nitrogen content is enhanced significantly. It is suggested that the nitrogen atoms retain in the condensed phase after cross-linking reaction.

**Fig. 11:**
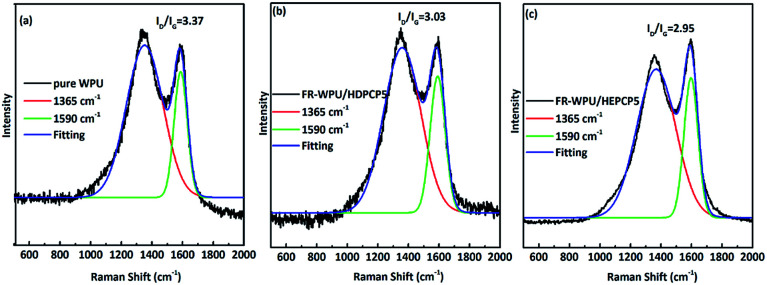
Raman spectra of the char residues: (a) pure WPU, (b) FR-WPU/HDPCP5 and (c) FR-WPU/HEPCP5.

To clarify the effect of HDPCP and HEPCP on the structure of the char layers, laser Raman spectroscopy was conducted to analyze the graphitization level of the residue of pure WPU, FR-WPU/HDPCP5 and FR-WPU/HEPCP5 from the CCT, as shown in Fig. 11. There were two strongly overlapping absorption peaks at around 1350 cm^−1^ (D band) and 1590 cm^−1^ (G band), which corresponded to the vibration of sp^2^-hybridized and the vibration mode with *E*_2g_ symmetry, respectively. Typically, the ratio of the integral area between the D and G bands (*I*_D_/*I*_G_) is an index to measure the degree of graphitization of char residues, and a higher *I*_D_/*I*_G_ value indicates a lower graphitization degree of char.^44^ The *I*_D_/*I*_G_s of pure WPU, FR-WPU/HDPCP5 and FR-WPU/HEPCP5 were 3.37, 3.03 and 2.95, indicating a high graphitization degree following the introduction of HDPCP or HEPCP. Char residue from FR-WPU/HEPCP5 had the lowest *I*_D_/*I*_G_ value. These results indicate that the incorporation of cyclotriphosphazene with a Schiff base facilitated the formation of a more stable char structure, with higher thermal stability and fewer defects. The carbonaceous layers that formed on the outer of polyurethane films limited both heat and flame transfer during combustion and showed a better flame retardant and smoke suppression performance. This result was in accordance with SEM analysis.

**Fig. 12:**
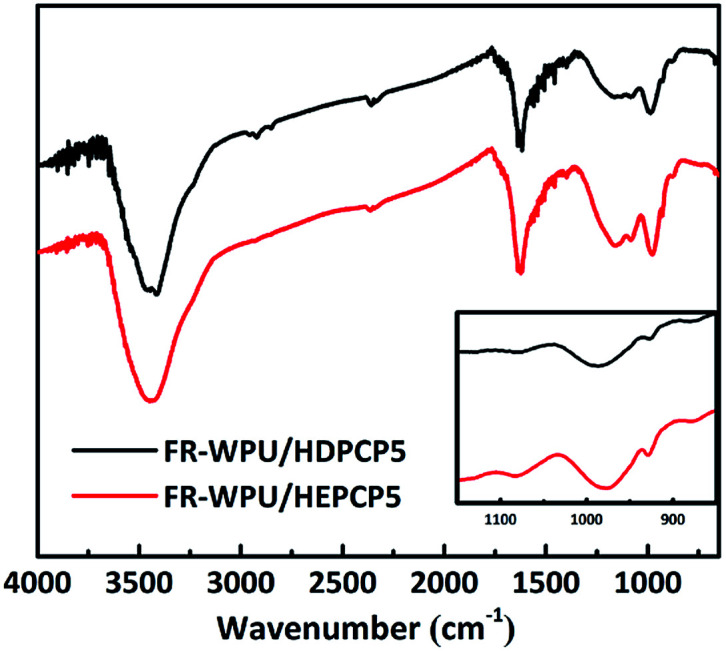
FT-IR spectra of the char residues.

The char residues of FR-WPU/HDPCP5 and FR-WPU/HEPCP5 from CCT were analyzed with FT-IR, and the results are shown in Fig. 12. The FT-IR spectra of two residual chars were almost identical. The significant signal at 3442 cm^−1^ corresponding to the fragments of –OH groups from absorbed water or –NH_2_ groups the in the solid phase;^46^ the peaks at 1624 cm^−1^ is attributed to the C=C bonds in primary amides; and the absorption signal at 1078 cm^−1^ indicates the presence of C–O group in chars. The absorption peak at 1163 cm^−1^ is attributed to C–N stretching vibration,^27^ which is an indication of incomplete combustion products in the condensed. It is caused during combustion by the hindrance of the formed char layer. In the case of FR-WPU/HEPCP5, the relative strength of absorption at 1163 cm^−1^ increased when compared with that in FR-WPU/HDPCP5. This change may also be caused by the crosslinking action of the azomethine double bond. The signals at 927 cm^−1^ and 983 cm^−1^ are attributed to P–O–C and P=N stretching vibration, respectively. The results indicate the presence of polyphosphate compounds. The FITR results of the FR-WPU/HDPCP5 and FR-WPU/HEPCP5 char residue further confirmed that both HDPCP and HEPCP mainly displayed a condensed phase mechanism.

HEPCP improved the flame retardancy of WPU, and mainly displayed a condensed phase mechanism *via* the formation of an intumescent char layer. The Schiff base and cyclotriphosphazene played a synergistic effect in WPU. The potential flame-retardant mechanism of the flame retardants is proposed to be as follows: first, the cleavages of phosphorus-containing groups with main-chain of polyurethane. Following this, the phosphorus-containing compounds in the matrix turn into polyphosphoric acids, causing dehydration and generating the carbonization layer. Finally, the char layers hinder the transfer of heat, oxygen diffusion and flammable gases. At the same time, the melting viscosity is increased by the crosslinking action of the azomethine double bond.

### Dynamic mechanical thermal analysis

**Fig. 13:**
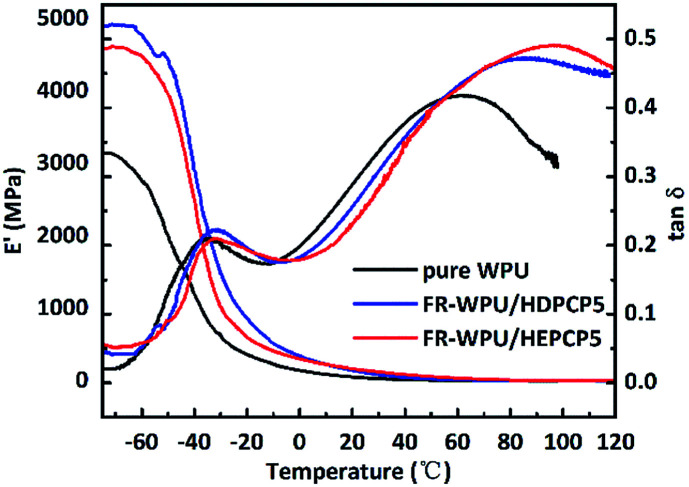
Storage modulus and tan *δ versus* temperature of pure WPU, FR-WPU/HDPCP5 and FR-WPU/HEPCP5.

DMA analysis was performed to determine the thermomechanical behavior of the pure WPU, FR-WPU/HDPCP5 and FR-WWPU/HEPCP5 films. The results of loss factor (tan *δ*) and storage modulus (*E*′) are shown in Fig. 13. The storage modulus of all the films was observed to suddenly drop at a temperature range of −75 °C to 20 °C, and then decrease slightly. This was likely due to thermal transitions that occurred in the WPU matrix. When HDPCP or HEPCP were incorporated into the WPU, a higher *E*′ was obtained. It is thought that the increase in *E*′ arose as a result of flame retardants that act as cross-linking agents to form a cross-linked net structure.^47–49^ The *E*′ of FR-WPU/HDPCP5 was higher than FR-WPU/HEPCP5, indicating that the rigid benzene ring restricted the vibrations and rotation of the polymer chain. In addition, as shown in Fig. 13, all the films showed two peaks in the tan *δ* curves, corresponding to the glass transition temperature of the hard segments (*T*_gh_) and the soft segments (*T*_gs_). The pure WPU had a *T*_gs_ value of −35 °C. After incorporating HDPCP or HEPCP, the *T*_gs_ value did not change significantly, although *T*_gh_ increased dramatically. For pure WPU, the *T*_gh_ value was 61.47 °C, whereas the *T*_gh_ value was 86.32 °C and 95.48 °C for FR-WPU/HDPCP5 and FR-WWPU/HEPCP5, respectively. The results demonstrate that both HDPCP and HEPCP increased the cross-linking densities of WPU.^50^

### Mechanical properties

**Fig. 14:**
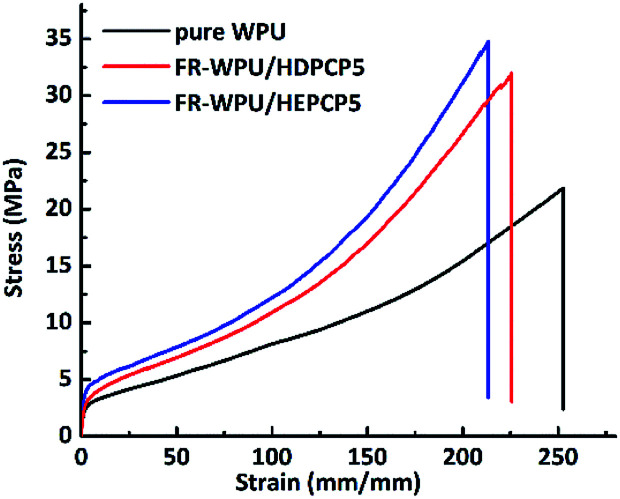
Stress–strain curves of pure WPU, FR-WPU/HDPCP5 and FR-WWPU/HEPCP5.

The mechanical properties of the pure WPU, FR-WPU/HDPCP5 and FR-WWPU/HEPCP5 films were investigated and are shown in Fig. 14. Data are given in Table 6. In case of pure WPU, the elongation at break, tensile strength and Young's modulus of pure WPU were 622%, 21.82 MPa and 6.09 MPa, respectively. After incorporating HDPCP or HEPCP into the WPU, the value of Young's modulus, tensile strength and elongation at break all increased. This may be because the moderation of crosslinking enhanced the effect of the flame retardants in the polymer matrix. In comparison with the FR-WPU/HDPCP5 films, the incorporation of HEPCP led to reinforcement tensile strength and Young's modulus on FR-WPU/HEPCP5 films. However, the elongation at break of the FR-WPU/HEPCP5 film decreased by 5.4% compared with that of the FR-WPU/HDPCP5 film. It is because of the rigid benzene ring units in HEPCP enhanced the degree of physical cross-linking, the phase separation degree of FR-WPU/HEPCP5 films increased, leading a remarkable improvement of the tensile strength and Young's modulus. According the results of DMA, the more difference in the glass transition temperature between the *T*_gh_ and *T*_gs_ the higher phase separation degree of FR-WPU films. At the same time, the steric hindrance derived from benzene ring units in HEPCP has impacted the molecular mobility of polyurethane chains. Hence, the elongation at break of FR-WPU films was decreased.

**Table 6:** Mechanical properties of pure WPU, FR-WPU/HDPCP5 and FR-WWPU/HEPCP5

Sample	Elongation at break (%)	Tensile strength/MPa	Young's modulus/MPa
Pure WPU-0	622	21.82	6.09
FR-WPU/HDPCP5	752	31.97	7.21
FR-WPU/HEPCP5	711	34.76	8.06

## Conclusion

HEPCP, a novel reactive intumescent fire retardant constructed with a Schiff base and cyclotriphosphazene structures, was utilized to prepare WPU. When HEPCP was incorporated into WPU, the FR-WPU/HEPCP5 exhibited a higher thermal stability and richer char residue under high temperatures, according to TG analysis. The FR-WPU/HEPCP5 possessed an LOI value of 26.7% and achieved a V-1 rating of UL-94 only by 0.5 wt% phosphorus-containing. In addition, the dripping behaviors were simultaneously improved. In a cone calorimetry test, the fire resistance of FR-WPU was remarkably enhanced. In contrast to pure WPU, the THR, pHRR, TSP and SPR of FR-WPU/HEPCP5 decreased by 23.24%, 43.8%, 41.26% and 63.94%, respectively. The results of TGA-FIR, Py-GC/MS and the morphology and chemical composition of residual char from CCT indicated that HEPCP displayed its fire-retardant mechanism in the condensed phase. The thermomechanical behaviors and the mechanical properties indicated that both the mechanical properties and *T*_gh_ increased.

## Conflicts of interest

There are no conflicts to declare.

## Supplementary Material
